# Association of serum high-mobility group box protein 1 level with outcomes of acute exacerbation of idiopathic pulmonary fibrosis and fibrosing nonspecific interstitial pneumonia

**DOI:** 10.1371/journal.pone.0196558

**Published:** 2018-05-24

**Authors:** Hiroshige Shimizu, Susumu Sakamoto, Takuma Isshiki, Kenta Furuya, Atsuko Kurosaki, Sakae Homma

**Affiliations:** 1 Department of Respiratory Medicine, Toho University Omori Medical Center, Ota-ku, Tokyo, Japan; 2 Department of Diagnostic Radiology, Fukujuji Hospital, Kiyose, Tokyo, Japan; University of Pittsburgh, UNITED STATES

## Abstract

**Background and objective:**

High-mobility group box 1 (HMGB1) protein is important in acute lung injury. However, the role of HMGB-1 in acute exacerbation of fibrosing interstitial pneumonia (AE-FIP) has not been adequately studied.

**Methods:**

We prospectively measured serum HMGB1 level from disease onset to day 7 in 36 patients with AE-FIP6 patients had missing data because of early death (within 7 days). We then examined the association of HMGB1 level and outcome, and the associations of rhTM with HMGB1 level and outcome in 19 patients who were treated with rhTM (rhTM group) and 11 patients who were not (control group).

**Results:**

Data from 36 AE-FIP patients (mean age, 73.5±6.7years) were analyzed. Serum HMGB1 level was significantly higher in patients with AE-FIP than in those with stable idiopathic pulmonary fibrosis (16.4±13.5 vs 5.7±2.6 ng/ml, respectively; p = 0.003). HMGB1 was significantly lower on day 7 than at AE-FIP onset in survivors (6.5±4.8 vs 14.7±12.9 ng/ml, respectively; p = 0.02) but not in nonsurvivors (14.6±10.5 vs 9.2±4.8 ng/ml, respectively; p = 0.08). Although HMGB1 level at day 7 was significantly lower after rhTM treatment than at AE-FIP onset (8.4±6.1 vs 15.2±12.5 ng/ml, respectively; p = 0.02), it did not significantly decrease in patients receiving treatments other than rhTM (11.3±11.3 vs 8.3±5.3 ng/ml, respectively; p = 0.37). Three-month survival was 60.0% in the rhTM group and 36.4% in the control group (p = 0.449). In multivariate analysis, a decrease in HMGB1 was a significant independent predictor of 3-month survival (Odds ratio, 12.4; p = 0.007).

**Conclusion:**

rhTM lowers serum HMGB1 level and may improve survival after AE-FIP. HMGB1 may be a promising therapeutic target for AE-FIP.

## Introduction

Idiopathic pulmonary fibrosis (IPF) is a chronic, fibrosing interstitial lung disease associated with the presence of a usual interstitial pneumonia (UIP) histologic pattern. The natural history is heterogeneous, and IPF may include periods of acute deterioration in respiratory function, which are termed acute exacerbations of IPF (AE-IPF) when a cause cannot be identified [1, 2]. AE-IPF is associated with high morbidity [3,4].

High-mobility group box 1 protein (HMGB1) is a nuclear protein that was originally defined as a transcription factor–like protein. HMGB1 is released from necrotic cells or actively secreted from activated macrophages, dendritic cells, and natural killer cells [5]. Receptor for advanced glycation end products (RAGE), toll-like receptor 2 (TLR2), and TLR4 are thought to be receptors for HMGB1 [6]. HMGB1 is important in the development of sepsis-associated acute lung injury (ALI) in mice and in humans with sepsis [7]. Ligation of HMGB1 to its receptors activates macrophages, thereby resulting in release of tumor necrosis factor α (TNF-α) and interleukin 1b. HMGB1 is a late mediator of sepsis-associated ALI and is associated with ALI prognosis [8].

Thrombomodulin (TM) is a transmembrane glycoprotein on the surface of vascular endothelial cells and is important in regulating intravascular coagulation. TM is composed of the active extracellular domain of thrombomodulin and forms a reversible complex with thrombin, thereby converting plasma protein C into activated protein C, which deactivates coagulant factors and the proinflammatory effects of thrombin. TM has unique anti-inflammatory properties in its N-terminal lectin-like domain, through binding and deactivating HMGB1 [9]. Recombinant human thrombomodulin (rhTM) is approved for treatment of disseminated vascular coagulopathy in Japan. Existing evidence suggests that disordered coagulation is important in IPF [10, 11], and increased HMGB1 levels were reported in bronchoalveolar lavage fluid from patients with AE-IPF [12]. Thus, decreasing HMGB1 expression might improve AE-IPF symptoms and outcomes by controlling the coagulation cascade and suppressing inflammation.

Several small-scale studies showed that rhTM was beneficial for AE-IPF [13–16]. The importance of HMGB1 in AE-FIP pathogenesis and its change over time and relation to rhTM treatment for AE- FIP are not well understood. Thus, we measured serum HMGB1 levels in patients with AE-FIP, stable IPF, and other ALI and in healthy controls. In addition, we assessed change in HMGB1 level from AE-FIP onset and the association of HMGB1 level with the outcome of rhTM treatment.

## Materials and methods

### Patients

We prospectively measured serum HMGB1 levels in 36 patients with AE-FIP who were admitted to Toho University Omori Medical Center during the period from January 2013 through August 2015. We assessed change in HMGB1 level from AE-IPF onset to day 7 by using an enzyme-linked immunosorbent assay (ELISA) for HMGB1 that included monoclonal antibodies to HMGB1, as previously described (HMGB1 ELISA Kit II, SHINO-TEST Corporation, Kanagawa, Japan) [17]. Serum samples were stored at a temperature of −70°C or lower until analysis of HMGB1.

**Fig 1:**
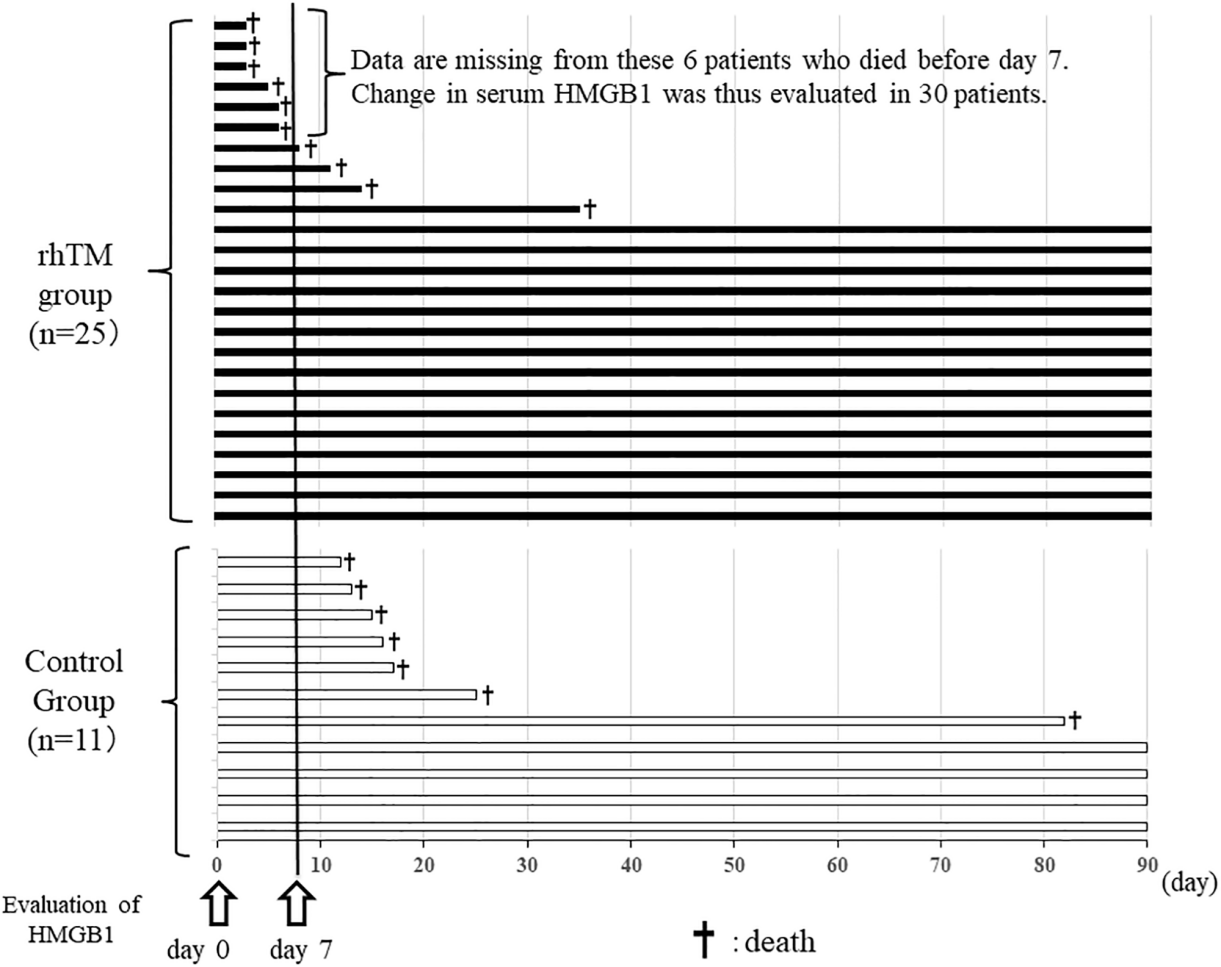
The clinical courses of all patients. Because six patients had missing data because of early death (within 7 days), change in serum HMGB1 was ultimately evaluated in 30 patients. An additional four patients in the rhTM group died during the period from day 7 through day 90. In the control group, no patient died within 7 days; however, seven patients died during the period from day 7 through day 90.

Although 36 patients who had received a first clinical diagnosis of AE-FIP were included in this study six had missing data because of early death (within 7 days). Ultimately, change in serum HMGB1 was evaluated in 30 patients. In addition, change in serum HMGB1 was compared between survivors (n = 19) and nonsurvivors (n = 11) at 3 months and between the 19 patients who received rhTM (rhTM group) and the 11 patients who did not (control group). We examined the effect of rhTM on HMGB1 level and its association with HMGB1 (Fig 1). In addition, we measured serum HMGB1 in persons with stable IPF (n = 15) and other ALI (n = 6) and in healthy participants (n = 5) and analyzed the associations of serum HMGB1 level with clinical variables in the patients with AE-FIP.

### Data collection

Clinical data were collected to determine the characteristics of underlying IPF and IPF treatment before AE. Specifically, we collected data on respiratory function during the 6-month period before AE and information on PaO_2_/FiO_2_ ratio, white blood cell count, C-reactive protein (CRP), lactate dehydrogenase (LDH), Krebs von den Lungen-6 (KL-6), surfactant protein D (SP-D), D-dimer, fibrin/fibrinogen degradation products (FDP), and brain natriuretic peptide (BNP) at AE onset.

### Diagnosis of FIP

In accordance with American Thoracic Society (ATS)/European Respiratory Society (ERS)/Japanese Respiratory Society (JRS)/Latin American Thoracic Association (ALAT) guidelines [4], we used high-resolution computed tomography (HRCT) images to diagnose definite and possible UIP patterns. In this study, FIP comprised both definite and possible UIP patterns on HRCT images.

### Diagnosis of AE-FIP

AE-FIP was defined by using previously reported criteria [1,2, 18], with slight modifications, as follows: (1) a previous or concurrent diagnosis of FIP, (2) unexplained acute respiratory deterioration, with a duration of generally less than 1 month, (3) new bilateral ground-glass opacities and/or consolidation superimposed on a background reticular or honeycomb pattern consistent with UIP pattern on HRCT imaging, (4) no evidence of pulmonary infection, and (5) deterioration not fully explained by cardiac failure, fluid overload, pulmonary embolism, or other possible causes of ALI. Left heart failure and pulmonary embolism were excluded by transthoracic echocardiography, test results for BNP and D-dimer, and contrast-enhanced CT. Using the classification of Akira et al [19], we classified the CT pattern of all patients at AE-IPF onset as diffuse, peripheral, or multifocal.

### Treatment of AE-FIP

**Fig 2:**
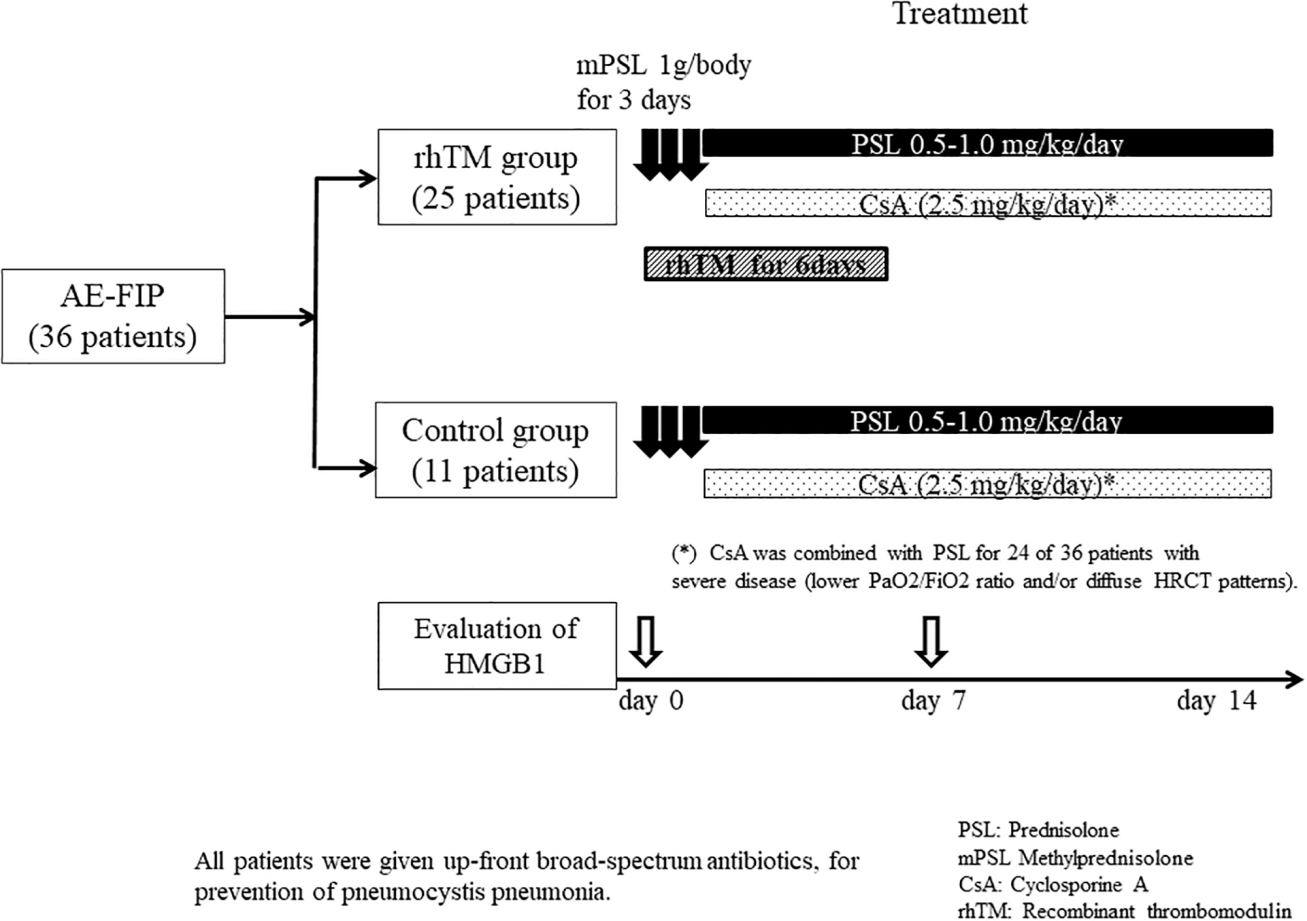
Study flowchart and treatment protocols.

All patients were treated with high-dose corticosteroid (CS) pulse therapy (methylprednisolone 1,000 mg/day for 3 days). CS dose was tapered after pulse therapy (0.5–1.0 mg/kg/day), and cotrimoxazole (a combination antibiotic containing trimethoprim and sulfamethoxazole) was prescribed for prevention of *pneumocystis* pneumonia. Cyclosporine A (2.5 mg/kg/day) therapy was combined with CS for 24 of the 36 patients with severe disease (lower PaO_2_/FiO_2_ ratio and/or diffuse HRCT patterns). In the rhTM group, rhTM was administered intravenously (diluted in 100 mL of sterile saline) at a dose of 0.06 mg/kg/day for the first 6 days, in combination with CS therapy. All patients received initial broad spectrum antibiotic therapy (Fig 2).

### Statistical analysis

Continuous variables are described as mean (±SD), unless otherwise stated, and were compared by using the Mann–Whitney U test. Categorical variables were compared with the χ^2^ test. Survival was analyzed with the Kaplan–Meier method, and differences were assessed with the log-rank test. Factors associated with 3-month survival were identified by univariate and multivariate logistic regression. A p value of less than 0.05 was considered to indicate statistical significance. All statistical analyses were performed with SPSS version 21.0 (IBM Corp).

### Ethics

This study was approved by the Institutional Review Board of Toho University *Omori* Medical Center (project approval number 23–168). All patients or their families provided written informed consent, and medical records were reviewed with the approval of the Institutional Review Board.

## Results

### Clinical characteristics of survivors and nonsurvivors

Table 1 shows the clinical characteristics of the survivors and nonsurvivors, at AE onset and before AE. There were no significant differences between groups in age, sex, PaO_2_/FiO_2_ ratio, estimated systolic pulmonary arterial pressure, D-dimer, KL-6, SP-D, or serologic markers at AE-IPF onset. The proportion of patients using cyclosporine A was significantly higher for survivors. HRCT imaging at AE-IPF onset showed diffuse ground-glass opacities and/or consolidation superimposed on preexisting subpleural fibrosis. The incidence of diffuse CT pattern was higher in nonsurvivors than survivors at AE-IPF onset.

**Table 1:** Clinical characteristics of survivors and nonsurvivors.

	Survivors (n = 19)	Nonsurvivors (n = 17)	p value
Age, yrs	73.9±6.6	73.1±7.1	0.74
Sex (male/female)	16/3	15/2	0.72
Brinkman index	678.4±436.8	741.2.0±663.4	0.74
P/F ratio	256.1±99.9	214.7±87.0	0.20
CRP (mg/dl)	6.6±5.1	10.5±7.0	0.06
APACHE II score	12.1±4.4	14.7±7.4	0.24
LDH (IU/l)	344.7±103.4	407.2±115.7	0.10
KL-6 (U/ml)	1895.2±2331.7	1346.6±700.5	0.46
SP-D (ng/ml)	375.1±319.7	547.9±339.1	0.13
D-dimer (μg/ml)	9.2±13.2	11.2±9.6	0.63
HMGB1, day 0 (ng/ml)	14.7±12.9	9.5±4.1	0.15
rhTM use (number [%])	15/19 (78.9%)	10/17 (58.8%)	0.19
CsA use (number [%])	17/19 (89.5%)	9/17 (52.9%)	0.02
HRCT pattern at the onset of AE Peripheral -multifocal/diffuse	13/6	6/11	0.04

CRP: C-reactive protein; LDH: lactate dehydrogenase; SP-D: surfactant protein-D; P/F ratio: PaO_2_ /FiO_2_ ratio, AE: acute exacerbation, CsA: Cyclosporine A

### Clinical characteristics of patients who were and were not treated with rhTM

Table 2 shows the clinical characteristics of patients who were and were not treated with rhTM, at AE onset and before AE. There were no significant differences between groups in age, sex, APACHE II score, PaO_2_/FiO_2_ ratio, estimated systolic pulmonary arterial pressure, D-dimer, KL-6, SP-D, cyclosporine A use, or serologic markers at AE-IPF onset. The diffuse CT pattern was the most frequent pattern in both groups at AE-IPF onset. Radiologic characteristics did not significantly differ between groups.

**Table 2:** Clinical characteristics of rhTM group and control group.

	rhTM group (n = 25)	Control group (n = 11)	p value
Age, yrs	73.1±6.8	74.5±6.8	0.59
Sex	22/3	9/2	0.49
Brinkman index	737.2±557.7	641.8±545.6	0.64
P/F ratio	227.5±102.2	257.3±76.7	0.67
CRP (mg/dl)	8.8±5.2	7.6±8.5	0.62
APACHE II score	14.4±5.5	12.6±3.9	0.48
LDH (IU/l)	387.5±113.9	344.0±107.8	0.29
KL-6 (U/ml)	1807.6±2077.7	1072.6±558.0	0.26
SP-D (ng/ml)	434.9±344.3	506.3±326.0	0.57
D-dimer (μg/ml)	9.5±10.4	11.4±14.6	0.67
HMGB1, day 0 (ng/ml)	14.4±11.5	8.3±5.3	0.11
CsA use (number [%])	17/19 (89.5%)	7/11 (63.6%)	0.69
HRCT pattern at the onset of AE Peripheral -multifocal/diffuse	14/11	5/6	0.72

CRP: C-reactive protein; LDH: lactate dehydrogenase; SP-D: surfactant protein-D; P/F ratio: PaO_2_ /FiO_2_ ratio, AE: acute exacerbation, CsA: Cyclosporine A

### HMGB1 level at AE-FIP onset

**Fig 3:**
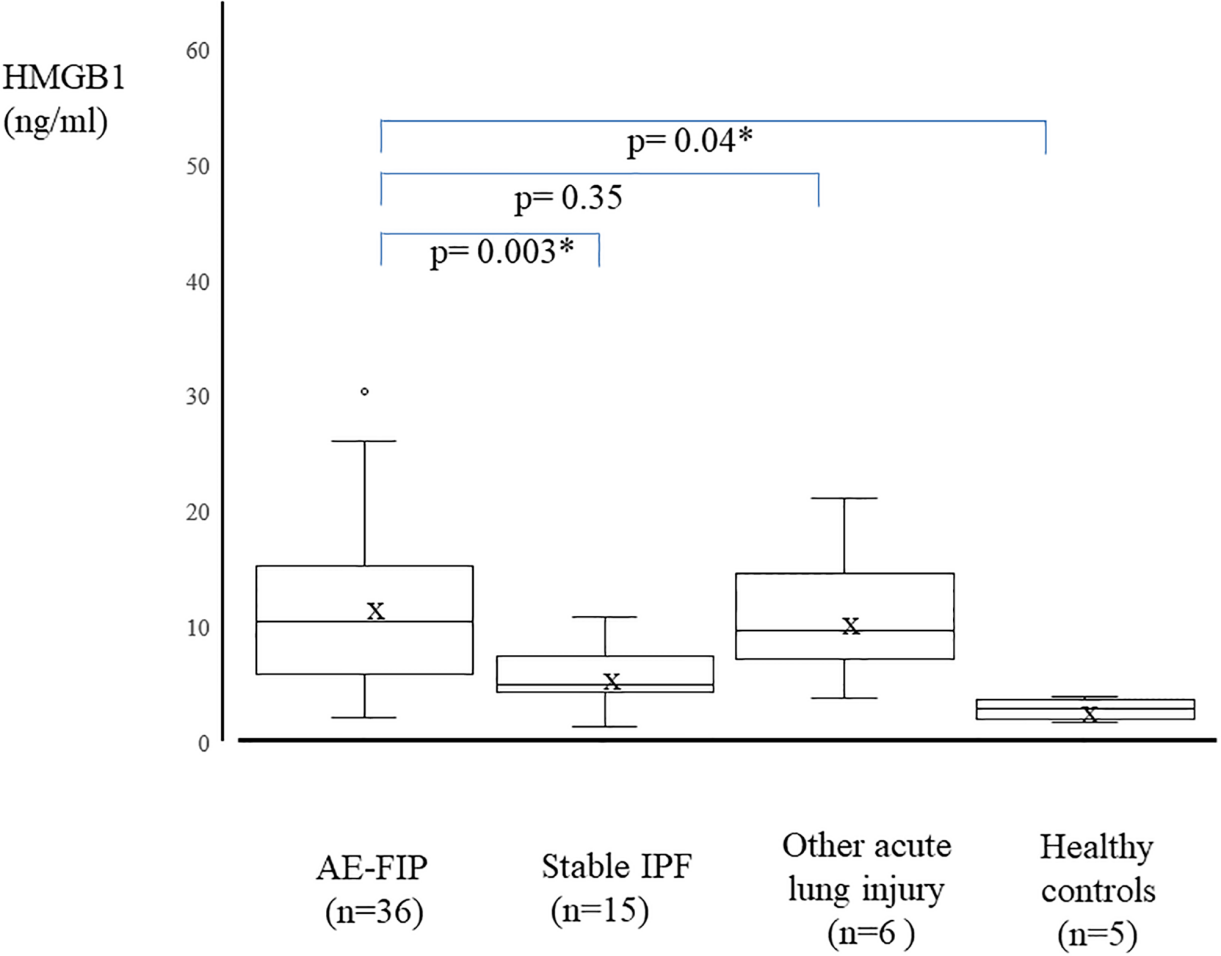
Serum high-mobility group box 1 protein (HMGB1) levels in patients with AE-FIP, stable IPF, and other acute lung injury, and in healthy controls. Whiskers show the minimum and maximum data values. Bars show median HMGB1 levels and the “x” indicates mean HMGB1 level. Serum HMGB-1 level was significantly higher in AE-FIP patients than in stable IPF patients and healthy controls.

Data from 36 FIP patients (mean age, 73.5±6.7 years) were analyzed. Serum HMGB1 level was significantly higher at AE-FIP onset than for healthy participants and patients with stable IPF (16.4±13.5 ng/ml vs 2.9±0.9 ng/ml and 5.7±2.6 ng/ml, respectively). HMGB1 was also higher in patients with other ALI, such as drug-induced pneumonitis and acute interstitial pneumonia. There was no significant difference in serum HMGB1 between patients with AE-FIP and those with ALI (16.4±13.5 vs 10.8±5.8 ng/ml, respectively). In addition, there was no significant difference in serum HMGB1 between patients with stable IPF and healthy participants (5.7±2.6 vs 2.9 ± 0.9 ng/ml, respectively; Fig 3). HMGB1 level was not associated with clinical variables such as LDH, CRP, KL-6, SP-D, SP-A, D-dimer, FDP, or P/F ratio (Table 3). In addition, there was no significant difference in serum HMGB1 at AE-FIP onset in relation to HRCT classification of AE-FIP (peripheral, multifocal, or diffuse pattern).

**Table 3:** Correlation between serum HMGB-1 levels and clinical parameters.

	p-value	correlation coefficient
HRCT diffuse pattern	0.206	-0.22
C-reactive protein	0.891	-0.02
Lactate dehydrogenase	0.766	-0.05
Krebs von den Lungen-6	0.620	-0.09
Surfactant protein D	0.063	-0.32
FDP	0.741	-0.06
D-dimer	0.618	-0.09
P/F ratio	0.628	0.09

FDP; fibrinogen degradation products

### Change in serum HMGB1 in survivors and nonsurvivors

**Fig 4:**
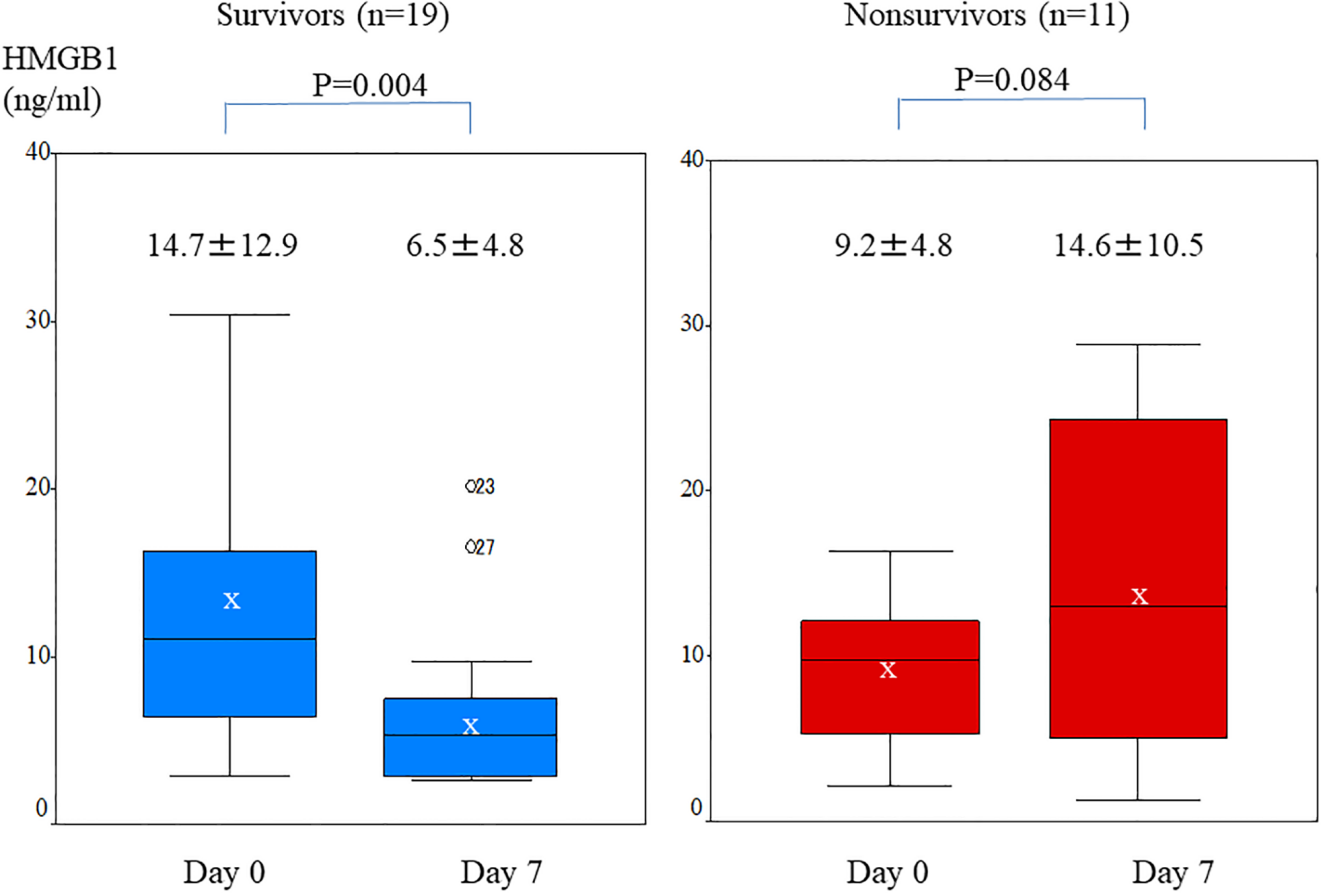
Change in serum HMGB1 level in survivors and nonsurvivors. Whisker showed the minimum and maximum of the data. Bars show median HMGB1 levels. The “x” indicates mean HMGB1 level. Among survivors, serum HMGB1 level was significantly lower on day 7 than on day 0 (6.5±4.8 vs 14.7±12.9 ng/ml, respectively; P = 0.004). In contrast, no significant difference in serum HMGB1 level was seen in nonsurvivors (14.6±10.5 vs 9.2±4.8 ng/ml, respectively; P = 0.08).

**Fig 5:**
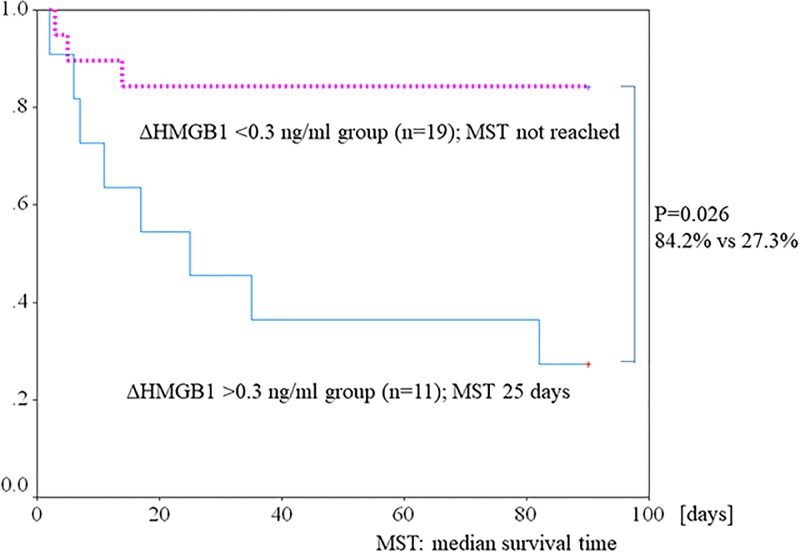
Kaplan–Meier survival curves for 3-month survival in the ΔHMGB1 <0.3 ng/ml group (n = 19) and ΔHMGB1 >0.3 ng/ml; group (n = 11). Survival at 3 months was significantly better in the ΔHMGB1 <0.3 ng/ml group (n = 19) than in the ΔHMGB1 >0.3 ng/ml group (n = 11) (84.2% vs 27.3%, respectively; p = 0.026). (MST: median survival time).

Fig 4 shows change in serum HMGB1. Although serum HMGB1 at onset did not significantly differ between survivors and nonsurvivors, serum HMGB1 level on day 7 was significantly lower in survivors than in nonsurvivors (6.5±4.8 vs 14.6±10.5, respectively; p = 0.006). Serum HMGB1 level was significantly lower on day 7 than on day 0 among survivors (14.7±12.9 vs 6.5±4.8 ng/ml, respectively; P = 0.004). In contrast, there was no significant difference between serum HMGB1 levels on days 0 and 7 among nonsurvivors (9.2±4.8 vs 14.6±10.5 ng/ml, respectively; P = 0.08. The decline in mean serum HMGB1 from day 0 to day 7 (expressed as ΔHMGB1) was significantly larger in survivors than in nonsurvivors (−8.22±10.79 vs 5.48±9.46, respectively; P = 0.003). Using these data, we evaluated the association of change in HMGB1 (ΔHMGB1) level with 3-month survival. We assessed the cut off value of ΔHMGB1 for survival using receiver operating characteristic curves. The optimal cutoff value for ΔHMGB1 was +0.3 ng/ml (area under the curve, 0.83). Fig 5 shows the 3-month survival curves for patients with a ΔHMGB1 level on day 7 that was >0.3 or <0.3 ng/ml higher than at baseline. Survival at 3 months was significantly better in the latter group (84.2%) than in the former group (27.3%) (p = 0.026)

### Change in serum HMGB1 in the rhTM group and control group

**Fig 6:**
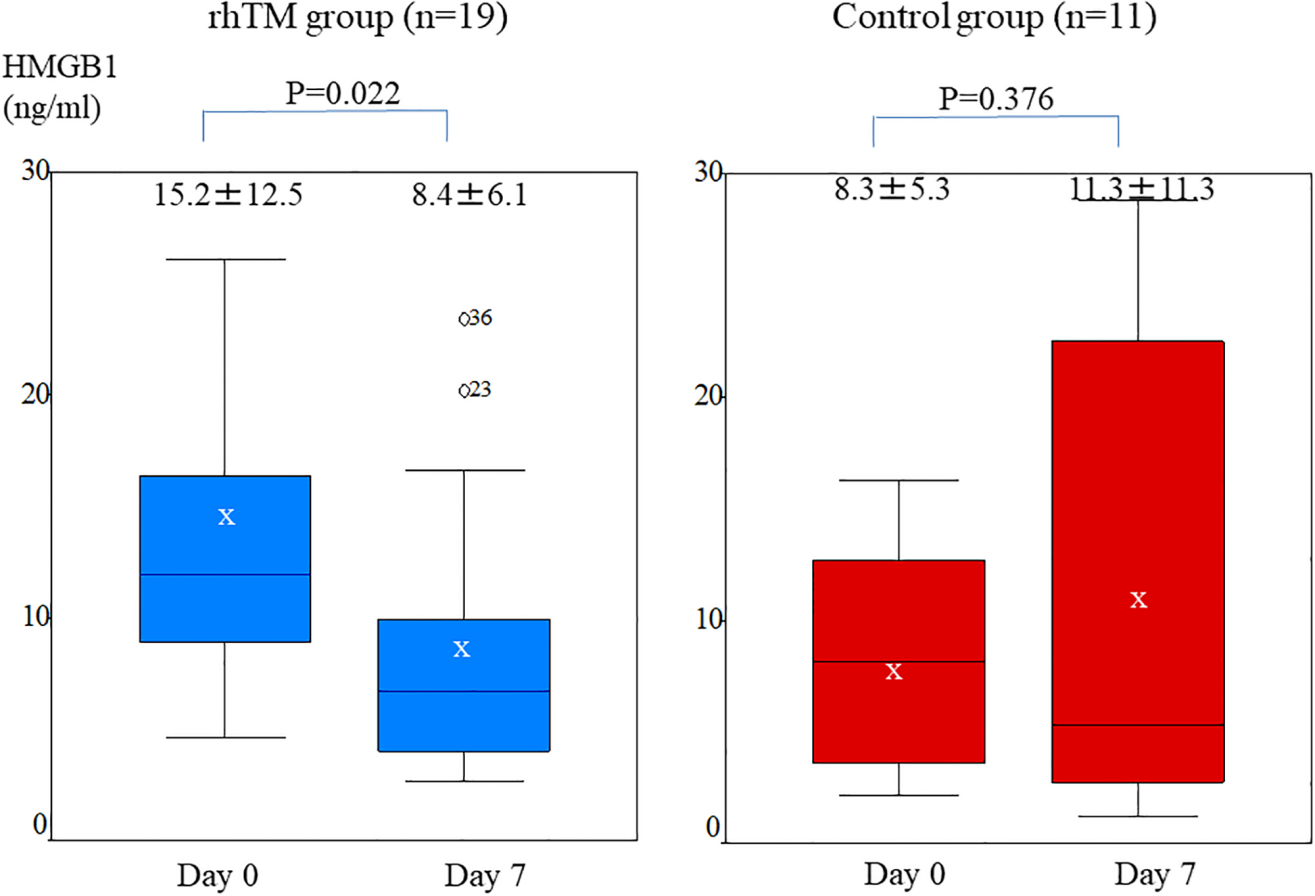
Change in serum HMGB1 level in the rhTM group and control group. Whiskers show the minimum and maximum data values. Bars show median HMGB1 levels, and “x” shows the mean HMGB1 level. At the 7-day follow-up examination, serum HMGB1 level was significantly lower than at onset of AE-FIP in patients treated with rhTM (8.4±6.1 vs 15.2±12.5 ng/ml, respectively; P = 0.02). In contrast, HMGB1 level did not significantly decrease in the control group (11.3±11.3 vs 8.3±5.3 ng/ml, respectively; P = 0.37).

**Fig 7:**
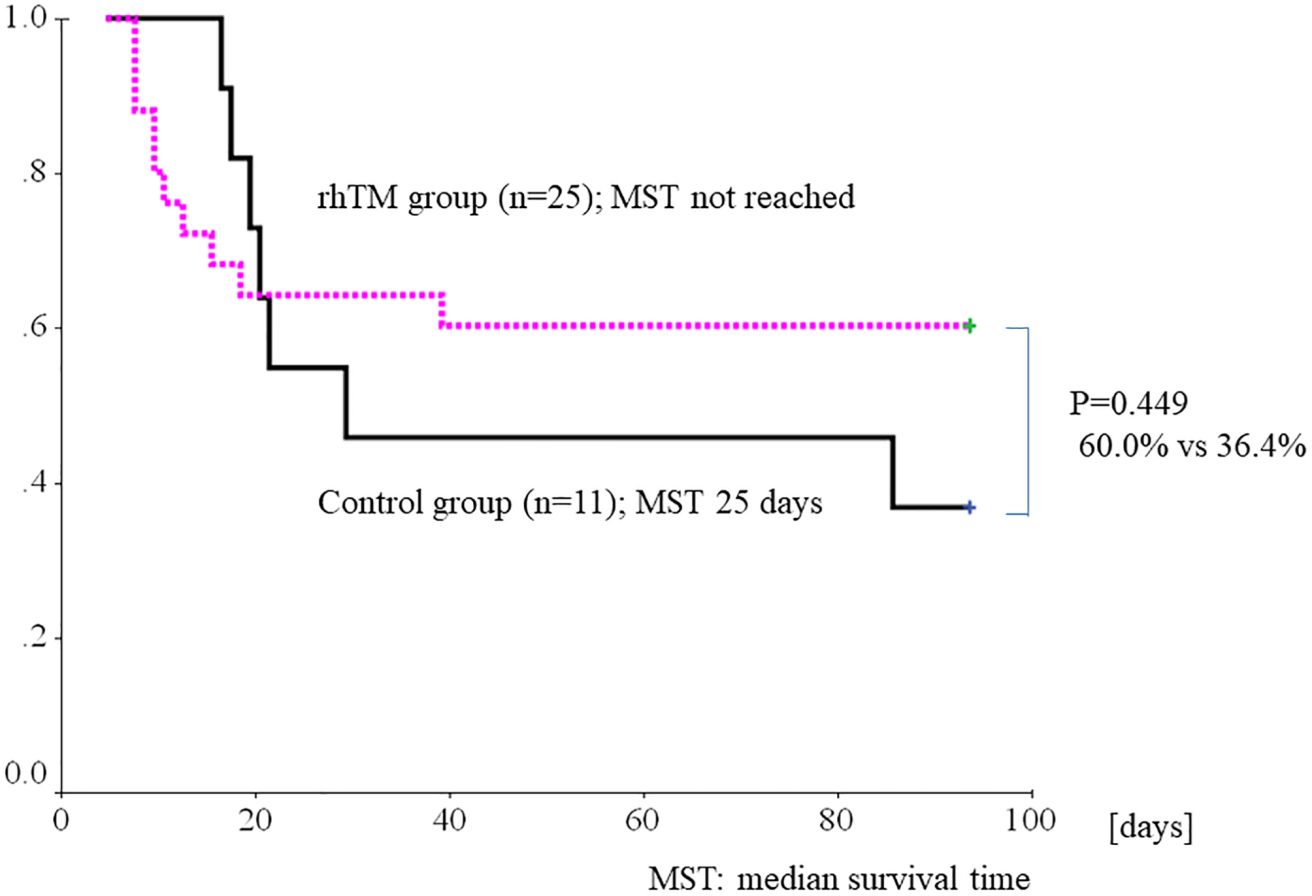
Kaplan–Meier survival curves for 3-month survival in patients treated with and without recombinant human soluble thrombomodulin (rhTM). There was no significant difference in survival at 3 months between the two groups (60.0% vs 36.4%, respectively; p = 0.449). (MST: median survival time).

At the 7-day follow-up examination, serum HMGB1 level was significantly lower than at AE-FIP onset in the rhTM group (8.4±6.1 vs 15.2±12.5 ng/ml, respectively; P = 0.02). In contrast, HMGB1 level did not significantly decrease in the control group (11.3±11.3 vs 8.3±5.3 ng/ml, respectively, P = 0.37; Fig 6). ΔHMGB1 decreased in the rhTM group but not in the control group, but the difference in ΔHMGB1 was not significant (−6.77±11.79 vs 2.98±10.63, respectively; P = 0.064). Fig 7 shows the 3-month survival curves for the rhTM and control groups. Survival at 3 months did not significantly differ between the two groups but was substantially better in the rhTM group than in the control group (60.0% vs 36.4%, respectively; p = 0.449).

### Factors predicting 3-month survival

During the observation period, 17 of the 36 patients (47.2%) died. All deaths during the first 3 months were from respiratory failure caused by AE-FIP. Univariate analysis showed that factors predicting 3-month survival were an HMGB1 level on day 7 that was <0.3 ng/ml higher than baseline Δ HMGB1 (odds ratio [OR], 14.2; 95% confidence interval [CI], 2.32–87.0; p = 0.004), and serum HMGB1 concentration on day 7 (OR, 0.873; 95% CI, 0.78–0.98; p = 0.019). Age, sex, other serologic markers (including KL-6 and SP-D), a diffuse CT pattern, D-dimer, pirfenidone use, and AE-IPF treatments other than rhTM were not prognostic factors. Multivariate analysis showed that 3-month survival was associated with a decrease in ΔHMGB1 (OR, 12.44; 95% CI, 2.00–77.6; p = 0.007; Table 4).

**Table 4:** Univariate and multivariate analysis of predictors of 3-month survival.

	p-value	Odds ratio	95% CI
	Univariate analysis		
Age	0.726	1.018	0.922–1.124
Male sex	0.728	0.711	0.104–4.864
Smoking index	0.168	3.542	0.586–21.397
P/F ratio	0.194	1.005	0.998–1.012
PaO_2_	0.316	1.032	0.970–1.097
D-dimer	0.621	0.985	0.929–1.045
FDP	0.842	0.997	0.968–1.027
White blood cell count	0.513	1.000	1.000–1.000
C-reactive protein	0.075	0.894	0.790–1.011
Lactate dehydrogenase	0.104	0.995	0.998–1.001
Krebs von den Lungen-6	0.470	1.000	1.000–1.001
Surfactant protein-D	0.132	0.998	0.996–1.001
Surfactant protein-A	0.967	1.000	0.986–1.013
HRCT diffuse pattern	0.051	0.252	0.063–1.008
HMGB1, day 0	0.176	1.086	0.964–1.223
HMGB1, day 7	0.019	0.873	0.779–0.978
Decline in HMGB1	0.004	14.222	2.324–87.028
rhTM use	0.197	2.625	0.606–11.372
	Multivariate analysis		
Decline in HMGB1	0.007	12.444	1.996–77.597

FDP; fibrinogen degradation products, rhTM; Recombinant human thrombomodulin

## Discussion

We investigated the role of HMGB1 in the pathogenesis of AE-FIP and found a significant difference in serum HMGB1 level between patients with AE-FIP and those with stable IPF and healthy controls. Serum HMGB1 level was significantly higher in patients with AE-FIP and ALI as compared with those with stable IPF and healthy controls. Abe et al showed that serum HMGB1 level was elevated at AE-IPF onset and decreased after treatment with a polymyxin B–immobilized fiber column [20].

Serum HMGB1 level was not associated with any clinical variable; however, change in HMGB1 was the only predictor of 3-month survival after AE-FIP. Gibot et al reported that serum HMGB1 level was correlated with sequential organ failure assessment (SOFA) score, lactates, and procalcitonin in patients with sepsis [21], which indicates that HMGB1 reflects systemic inflammation. HMGB1 was originally defined as a transcription factor–like protein and is thought to be a cytokine-like molecule. Therefore, HMGB1 has multiple functions in infection, tissue injury, inflammation, apoptosis, and immune response. Moreover, an increase in HMGB1 activated transforming growth factor β1 signaling and matrix metalloproteinase 9 in a murine model of bleomycin-induced IPF, which indicates that inflammation caused by HMGB1 promotes fibrosis [22, 23].

Limited clinical evidence indicates that although serum HMGB1 level was significantly higher in AE-IPF patients than in those with stable IPF, serum HMGB1 level at onset did not predict survival [15, 20]. Similarly, HMGB1 levels in other systemic inflammatory disorders, such as acute respiratory distress syndrome or sepsis, at disease onset did not predict survival [21, 24]. Gibot et al showed that, among patients with sepsis, serum HMGB1 was significantly lower in survivors than in nonsurvivors [23].

Hamada et al reported that HMGB1 was predominantly localized in nuclei of infiltrating inflammatory cells, alveolar macrophages, and epithelial cells in affected IPF lesions and noted positive staining in cytoplasm. These results suggest that HMGB1 expression is up-regulated in affected lesions and that secreted HMGB1 has a role as a proinflammatory cytokine in IPF, as was reported in a study using a murine model of bleomycin-induced pulmonary fibrosis. HMGB1 in the cytoplasm of epithelial cells and macrophages appeared to contribute to ALI and suppress subsequent fibrosis in that model [25].

Kim et al reported that anti-HMGB1 antibody prevented nuclear factor-κB (NF-κB) activation, proinflammatory cytokine production, and lung permeability in ALI [26]. Furthermore, Hamada et al found that HMGB1 directly stimulates fibroblast survival and collagen synthesis in vitro. In addition, they found that HMGB1 induced proliferation of human fibroblasts. Their results suggest that, in addition to its role as a trigger of inflammation, HMGB1 directly stimulates fibroblast proliferation and participates in fibrogenesis.

Ulloa et al reported that ethyl pyruvate, which inhibits release of HMGB1 from activated macrophages, inhibited release of TNF-α and HMGB1 from macrophages by interfering with activation of the p38 MAPK and NF-κB signaling pathways. They also noted that ethyl pyruvate treatment prevented lethal sepsis in animals [27]. Hamada et al demonstrated that ethyl pyruvate significantly reduced HMGB1 levels and the numbers of all cells, lymphocytes, and neutrophils in bronchoalveolar lavage fluid in their bleomycin mouse model [25]. Although it is not clear whether the attenuation observed in this model was caused by inhibition of HMGB1 release from macrophages or direct inhibition of the p38 MAPK and NF-kB signaling pathways, these results suggest that, in addition to inhibiting lethal sepsis in an in vivo model, anti-HMGB1 treatment might inhibit ALI and suppress subsequent fibrosis.

In this study, decreased serum HMGB1 was a significant independent predictor of 3-month survival (hazard ratio, 0.78; p = 0.03). Moreover, serum HMGB1 level significantly decreased after AE-FIP in the rhTM-treated group but not in patients who received treatments other than rhTM. These results support the hypothesis that rhTM treatment lowers HMGB1 level and improves survival after AE-FIP. Thus, HMGB1 is a promising therapeutic target for AE-FIP.

Inflammation and vascular injury, including loss of epithelial cell integrity, have been reported in interstitial pneumonias. Thus, thrombosis may also be present in pulmonary vasculature. In addition, the coagulation cascade is thought to precede inflammatory and fibroproliferative responses. Coagulation, inflammation, fibroproliferation, and tissue remodeling associated with the normal wound healing response may, because of repeated tissue injury or an aberrant repair mechanism in lungs affected by IPF, result in excess deposition of extracellular matrix proteins [28].

Although the pathophysiology of AE-IPF is not well understood, evidence form several studies suggests that disordered coagulation is important. Collard et al reported significant elevations in plasma biomarkers of endothelial cell injury and coagulation in AE-IPF patients. In addition, serum thrombomodulin level was a significant prognostic marker [10].

rhTM activates protein C, which deactivates coagulant factors and the proinflammatory effects of thrombin. Activated protein C also exerts anti-inflammatory effects by downregulating expression of inflammatory cytokines such as TNF-α. Moreover, the N-terminal lectin-like domain of rhTM deactivates HMGB1, which has an anti-inflammatory effect. Elevation of serum HMGB1, which is released from necrotic cells and subsequently causes cell damage, was observed in patients with sepsis [29] and ALI [30]. Furthermore, HMGB1 was elevated in bronchoalveolar lavage fluid from AE-IPF patients [12]. rhTM treatment is thus likely to benefit AE-IPF patients.

Previous studies reported a 3-month survival rate of 30% to 40% after AE-IPF onset in patients receiving conventional CS treatment [1–2, 31–33]. The survival rate of the present control group given the usual treatment was within this range. Although survival at 3 months did not significantly differ between the two groups, it was substantially better in the rhTM group than in the control group (60.0% vs 36.4%). These results are consistent with those of earlier studies, which showed improved 3-month survival after rhTM treatment for AE-IPF [13–16]. Thus, past and present evidence suggests that intravenous rhTM treatment may help improve outcomes for AE-IPF patients.

### Limitations

This study has several limitations. First, it was a single-center study of a relatively small number of patients. Because the number of patients was small, it remains to be determined whether rhTM directly decreases HMGB1 in patients with AE-FIP or if serum HMGB1 level is merely a prognostic marker for these patients. Our data show that serum HMGB1 was significantly lower and survival was better in the rhTM-treated group than in the control group. Larger-scale studies should be conducted in order to confirm our findings. Second, bronchoscopy could not be performed because of the presence of severe respiratory failure. Infection was ruled out in all cases by using less invasive procedures such as sputum and/or blood culture, urinary antigen tests, and serologic analysis. However, we cannot completely exclude the possibility of coexisting infection. Third, as we could not assess change in serum HMGB1 in patients who died within 7 days, there is a possibility of bias in the results. Finally, serum HMGB1 did not decrease in all patients receiving rhTM in this study, which suggests that rhTM improves survival by multiple mechanisms. Because the pathophysiology of AE-IPF is complex and multifactorial, HMGB1 level only partially reflects this condition. The other mechanisms that contribute to AE-IPF remain to be determined.

## Conclusion

In conclusion, serum HMGB1 level was significantly higher in patients with AE-FIP than in healthy controls and those with stable IPF. A decline in serum HMGB1 level during the period from onset to day 7 was a predictor of 3-month survival. rhTM treatment may lower serum HMGB1 level and deactivate coagulant factors, thereby possibly improving survival after AE-FIP. Although the precise role of HMGB1 in AE-FIP is unknown, HMGB1 inhibition might be an effective strategy against AE-FIP. Therefore, HMGB1 is a promising therapeutic target for AE-FIP.

## Supporting information

S1 Fig Raw clinical data. (XLSX)
